# Corrigendum: A Detailed View of KIR Haplotype Structures and Gene Families as Provided by a New Motif-Based Multiple Sequence Alignment

**DOI:** 10.3389/fimmu.2021.724357

**Published:** 2021-07-01

**Authors:** David Roe, Cynthia Vierra-Green, Chul-Woo Pyo, Daniel E. Geraghty, Stephen R. Spellman, Martin Maiers, Rui Kuang

**Affiliations:** ^1^ Bioinformatics and Computational Biology, University of Minnesota, Rochester, MN, United States; ^2^ Center for International Blood and Marrow Transplant Research, Minneapolis, MN, United States; ^3^ Clinical Research Division, Fred Hutchinson Cancer Research Center, Seattle, WA, United States; ^4^ Department of Computer Science and Engineering, University of Minnesota, Minneapolis, MN, United States

**Keywords:** killer-cell immunoglobulin-like receptors (KIR), alignment, DNA, haplotype, nomenclature, natural killer

There is an error in the Funding statement. In the original article, **OT3HL147741** was erroneously included as a funding source.

The authors apologize for this error and state that this does not change the scientific conclusions of the article in any way. The original article has been updated.

