# Supplementary figures and images for: A Detailed View of KIR Haplotype Structures and Gene Families as Provided by a New Motif-Based Multiple Sequence Alignment
Source: Front Immunol. 2020 Nov 18;11:585731. doi: 10.3389/fimmu.2020.585731 (PMC7708349; doi:10.3389/fimmu.2020.585731)

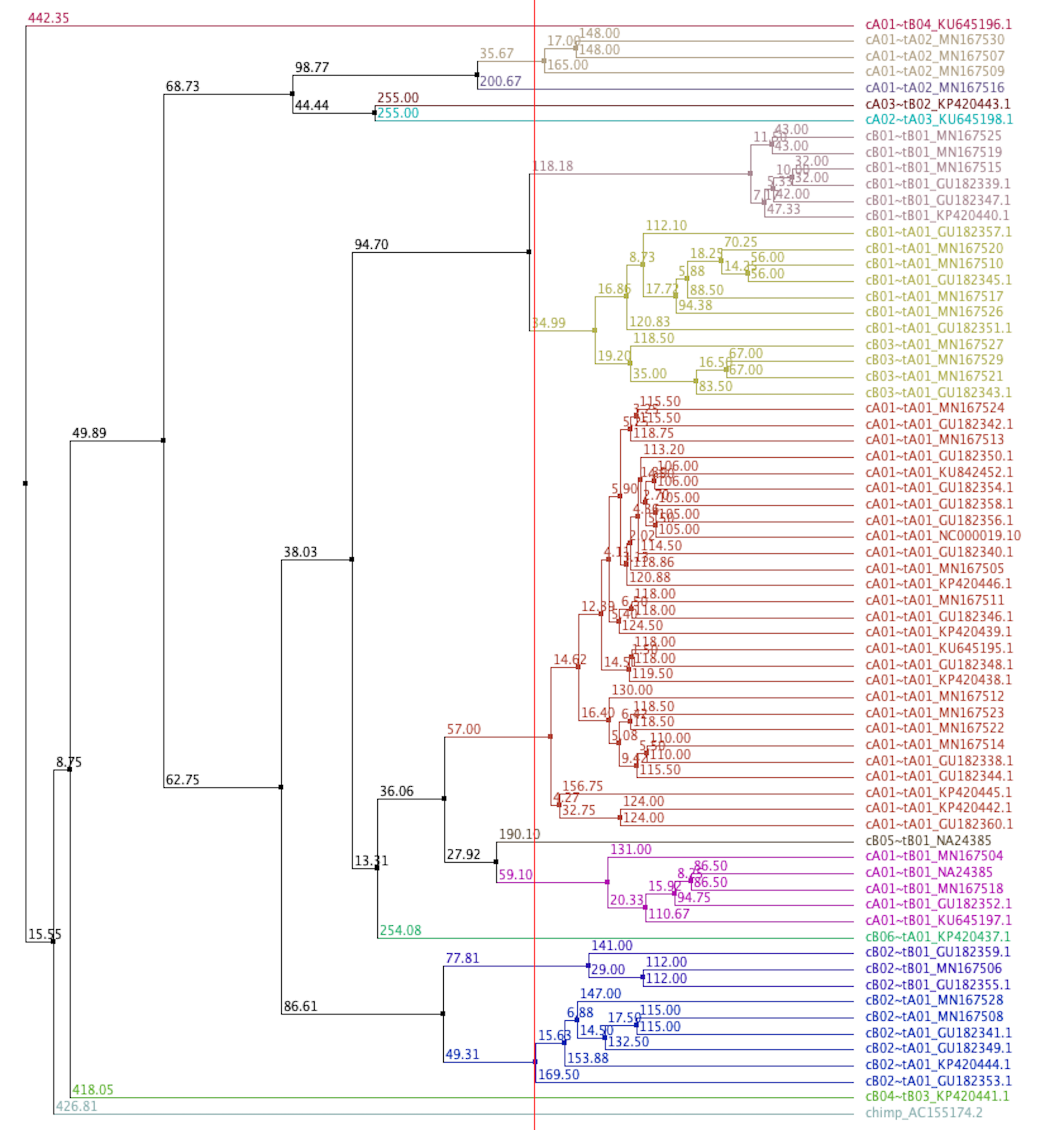

Supplement: Supplementary Figure 1 — Phylogenetic tree made from the phylogenetic tree of the probe motifs of 68 human haplotypes and 1 chimpanzee in Supplemental Data Sheet 2. The relatively short chimpanzee haplotype is an outlier on the bottom of the tree and the relatively long cA01~tB04 is an outlier on the top. [file Image_1.tiff]

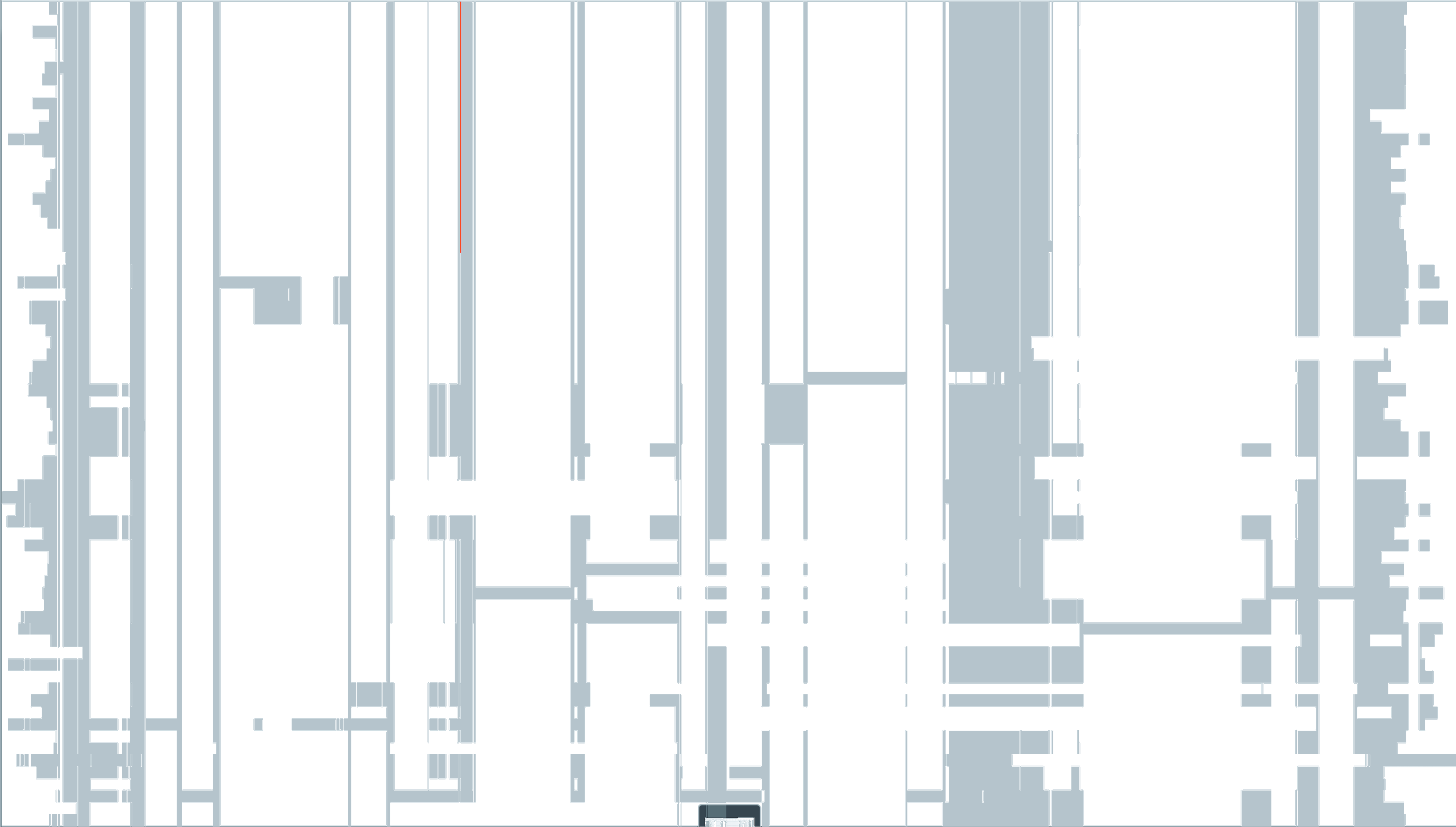

Supplement: Supplementary Figure 2 — Jalview overview of the MAFFT-generated multiple sequence alignment of the DNA of 68 human haplotypes plus 1 chimpanzee (Supplementary Data Sheet 4). [file Image_2.tiff]
